# Drinking Motives and Alcohol Consumption Among Asian American Young Adults: The Moderating Role of Alcohol-Related Facial Flushing

**DOI:** 10.3390/ijerph22111604

**Published:** 2025-10-22

**Authors:** Karen G. Chartier, Benjamin N. Montemayor, Jacyra de Araujo, Arham Hassan, on behalf of the Spit for Science Working Group

**Affiliations:** 1 School of Social Work, Virginia Commonwealth University, 1000 Floyd Avenue, Richmond, VA 23284, USA; araujoj3@vcu.edu; 2 Virginia Institute for Psychiatric and Behavioral Genetics, Virginia Commonwealth University, 800 East Leigh Street, Richmond, VA 23298, USA; 3 Department of Health Behavior, School of Public Health, Texas A&M University, College Station, TX 77843, USA; bnmontemayor@tamu.edu (B.N.M.); arham.hassan@tamu.edu (A.H.); 4 Cohort and Registry Administration Core, Virginia Commonwealth University, 800 East Leigh Street, Richmond, VA 23298, USA; spit4science@vcu.edu

**Keywords:** alcohol consumption, young adults, Asian American health, facial flushing, drinking motives, peer drinking, interaction effects

## Abstract

Background: Despite guidelines recommending lower alcohol limits for individuals who flush, some still drink at unhealthy levels. This study investigates whether drinking motives are differentially associated with alcohol consumption based on self-reported flushing status among U.S. Asian young adults. Asian American youth report alcohol use at rates comparable to other high-risk groups, identifying the need to understand factors shaping these behaviors. Methods: The current analysis drew participants from a longitudinal multi-cohort study examining the emotional and behavioral health of college students. Freshmen were recruited, all aged 18 years and older, to complete a baseline survey and follow up surveys over a four-year period. The analytic sample (Mean age = 19.4; 70.5% female) included 244 students who self-identified as Asian. Participants self-reported whether they experience facial flush when consuming alcohol and rated their endorsement of various drinking motives. Negative binomial regression models tested main effects and interaction effects between flushing status (flushers, non-flushers) and drinking motives (coping, enhancement, conformity, social). Results: Facial flushing moderated enhancement, conformity, and social drinking motives, but not coping. Among flushers, enhancement and social motives were more strongly associated with greater alcohol consumption. Among non-flushers, conformity motives were stronger and associated with greater drinking, at a trend level. Overall, flushing or higher coping motives were associated with lower alcohol consumption. Peer drinking was associated with higher consumption in both flushing-status groups. Conclusions: The current study extends prior international research on drinking motives and flushing status to U.S. Asian young adults. Findings support the need for prevention strategies that address individual drinking motives and the modeling of alcohol use by peers. Reducing alcohol use among individuals who experience alcohol-induced flushing is a public health priority, given their heightened risk for alcohol-related cancers and other negative health outcomes.

## 1. Introduction

Over the past two decades, from 2000 to 2020, Asian Americans were the fastest-growing racial and ethnic group in the U.S., nearly doubling from 10.5 to 19 million [1]. Despite this growth, their alcohol use patterns and associated risks remain underrepresented in the literature. Asian Americans are identified by the U.S. National Institute on Minority Health and Health Disparities (NIMHD) as a health disparity population, due to ongoing gaps in research and access to culturally relevant prevention services. This designation supports the need for more focused inquiry into their health behaviors [2]. Although Asian Americans have been perceived as a low-risk group for alcohol misuse, recent national data dispel this assumption. Between 1992 and 2001, alcohol use disorder (AUD) diagnoses among Asian Americans increased fivefold, and from 2001 to 2013, past-year alcohol use rose from 48.4% to 62.5%, the largest increase among all racial and ethnic groups [3,4]. Alarmingly, Asian American young adults (ages 18–25) report rates of past-month alcohol use and unhealthy drinking, comparable to rates observed in such high-risk groups as White and Greek-affiliated college students [5,6]. Additionally, international trends show increased alcohol use and AUD diagnoses among alcohol-intolerant individuals [7]. Alcohol-related harms are particularly pronounced among Asian American college students [8], and prior reviews have highlighted the need for more research on Asian American drinking behaviors, including high-risk subgroup differences within this population [9].

While research has documented rising alcohol use among Asian Americans, limited studies have examined the factors influencing these behaviors, particularly in U.S. college environments. Prior work has often generalized findings across ethnic and racial groups without considering cultural or physiological differences that influence drinking behaviors. This lack of targeted research overlooks important nuances, such as the role of biology (e.g., genetic variations related to alcohol intolerance), or psychosocial factors (e.g., identity-based drinking motives) unique to Asian American students [10,11]. Additionally, few studies have focused on how these students interpret and respond to alcohol-related symptoms known to influence the drinking behaviors of individuals from Asian descent, such as strategies to hide facial flushing, in drinking situations [12,13]. A more nuanced understanding of these factors and their interrelationships is essential for informing culturally responsive prevention strategies. The continued aggregation of data and absence of tailored research efforts may obscure within-group discrepancies and contribute to disparities in alcohol-related outcomes [14].

A key factor influencing alcohol use among Asians is alcohol intolerance, caused by the ALDH2*2 (rs671), ADH1B*2 (rs1229984), and other alleles for alcohol metabolism [15]. These genetic variants impair the metabolism of acetaldehyde, a toxic byproduct of alcohol, leading to intense physiological reactions such as facial redness/flushing, nausea, and rapid heartbeat that affect between 20% and 47% of individuals of Asian ancestry [16]. Alcohol is a leading cause of death and disability globally, responsible for 3 million deaths each year, and disproportionately affecting young people [17]. The public health impact is especially pronounced among individuals with alcohol-induced flushing [18]. Beyond discomfort, these responses signal elevated risks for serious health outcomes associated with drinking alcohol such as esophageal cancer, hypertension, and cardiovascular disease [19,20,21], with U.S. representative data revealing that Asians, particularly women and those from subgroups with higher prevalence of ALDH2*2, face increased risk of chronic health conditions even at relatively low levels of alcohol consumption [20]. Alcohol-related health risks among Asian populations are further compounded by tobacco co-use [22], although whether those who flush use tobacco at higher or lower rates than non-flushers may depend on the population surveyed [23,24]. Still, smoking frequently co-occurs with alcohol consumption among Asian college students [10], making it a lifestyle factor to consider when assessing alcohol use and flushing. Studying alcohol use and related factors in this population offers a critical lens into how biological vulnerability interacts with behavioral risk. While facial flushing has been traditionally seen as protective against unhealthy drinking [19], research indicates that this protective effect diminishes in peer-driven environments, including for young adults in college settings, where social norms may override discomfort [25,26].

Guidelines recommend lower drinking limits for people who flush when consuming alcohol [27], yet, some individuals still exceed these recommendations [7,28,29]. One possible explanation could be that they interpret flushing symptoms as manageable or even ignore them in order to maintain social connections or avoid stigma [30]. As such, further study of drinking motives may better inform our understanding of the reasons for consuming alcohol by this unique subgroup [31,32]. This is especially important from a public health perspective, where identifying modifiable mechanisms, such as drinking motives, can play a key role in reducing alcohol consumption and improving the effectiveness of screening and other prevention efforts [18,19], rather than looking to understand alcohol use behaviors more generally. Drinking motives, those are, motives focused on coping, enhancement, conformity, and social factors, are key predictors of alcohol use behaviors [33]. Drinking motives are commonly grouped into two broad categories: affect regulation motives, which encompass enhancement and coping motives; and social motives, which include both social and conformity-related reasons for drinking [34,35]. Enhancement motives reflect a desire to achieve positive emotions or experiences from drinking alcohol [36], and coping motives involve using alcohol to alleviate stress or negative emotions [37]. Social motives, which involve drinking to enhance social interactions and fit in with peers, are particularly prevalent in college settings [38]. Conformity motives, on the other hand, can stem from peer pressure or the fear of social exclusion, including modern concepts like fear of missing out (FOMO) [39]. Generally, social and enhancement motives are linked to greater alcohol consumption, while coping and conformity motives are associated with higher risks of alcohol-related problems [40,41].

Few studies have explored subgroup differences in drinking motives, such as variations across U.S. racial and ethnic groups or within Asian American subgroups. For example, LaBrie et al. [42] found that Asian American students were more likely to endorse coping and conformity motives, whereas White students more often reported enhancement motives. Research focused specifically on U.S. Asian young adults suggests that drinking motives evolve over time and vary by drinking behavior patterns. Among Asian American college students, motives related to having fun or being social, as well as relaxing or coping, tended to increase across semesters, while motives to avoid negative consequences declined [43]. Additionally, individuals who rated fun/social motivations as their most important motive drank more frequently and in greater quantities [43]. Iwamoto et al. [44] further found that greater endorsement of coping motives was linked to membership in high-risk binge drinking and monthly binge drinking classes, compared to a no-binge drinking class. However, these studies did not compare the drinking motives of people who do and do not experience alcohol-related flushing, which is a known key-factor in shaping the drinking patterns of individuals of Asian descent.

The relationship between drinking motives and alcohol sensitivity factors such as facial flushing remains largely underexplored in the existing literature, particularly in U.S. populations. Kim et al. [45] used focus groups to explore motivational factors for moderate drinking among young Asians in Singapore with varying flushing experiences (e.g., current flushers, former flushers, and non-flushers). Their findings suggested that flushers were more likely to overestimate peer influences and perceive greater social pressure to drink larger amounts of alcohol compared to non-flushers. Oh et al. [26] observed few overall differences in the drinking motives of flushers and non-flushers in South Korea; both groups commonly drank alcohol for pleasure or to relieve stress or depression, though boredom emerged as a motive primarily among non-flushers. When further stratified by sex, female flushers primarily drank for pleasure while male flushers drank due to stress or depression [26]. Both male and female non-flushers reported drinking primarily for pleasure. Yet, these studies on drinking motives, flushing, and alcohol consumption were conducted outside the U.S., and their findings may not fully generalize American college students.

Given the unique social and cultural factors that influence drinking behaviors in U.S. college environments, the present study seeks to examine how experiencing facial flushing interacts with drinking motives to shape alcohol consumption among a sample of Asian students at an American university. We hypothesize that flushing status will moderate relationships between drinking motives (coping, enhancement, conformity, and social factors) and alcohol consumption. Although perceived facial flushing has not been studied in this way, prior research on *ALDH2* (rs671) genotypes provides evidence of moderation effects [32]. By examining the interplay between drinking motives and self-reported flushing, this study aims to clarify how physiological sensitivity to alcohol influences drinking motives and alcohol use behaviors. In addition, we examine peer influences, mental distress, and current smoking status given their documented relevance to alcohol use among flushers and non-flushers [20,26,45,46]. Findings from this research may offer insights for developing interventions to reduce alcohol-related harms in a population that is highly susceptible to the effects of unhealthy alcohol use [18]. Understanding the different motivations that may diminish or enhance the protective effects of flushing against drinking can inform targeted prevention interventions, contribute to more effective and collaborative campus health programming, and advance public health guidance tailored to this high-risk group.

## 2. Methods

### 2.1. Sample

Data for this study were drawn from Spit for Science [47], a longitudinal research project started in 2011 at a U.S. mid-Atlantic public university, aimed at understanding how genetic, environmental, and developmental influences contribute to emotional and behavioral health outcomes during the college years. To date, the parent study includes eight cohorts. When a new cohort is launched, all incoming freshmen (18 years and older) receive an email invitation to participate. Students who consent, complete a self-administered baseline survey during their first year and are invited to complete follow up surveys over the next three years, provided they’re enrolled at the university. Survey data are collected online and managed through REDCap [48]. The study was reviewed and approved by the university’s Institutional Review Board.

The analytic sample consisted of a total of 244 participants from cohorts 3–5 (first-semester freshmen in 2013, 2014, and 2017). Students were included in the sample if they self-identified as Asian and provided data on alcohol-induced facial flushing. Those students who reported never drinking alcohol were excluded (as they were not asked questions about facial flushing). Students from other racial and ethnic groups represented in the parent study were also excluded, due to the low frequency of ALDH2*2 and ADH1B*2 in those populations. Study variables primarily came from the participants’ sophomore-year spring responses, with exception for time invariant variables such as race and ethnicity, sex, and facial flushing.

### 2.2. Measures

Demographics. Sample demographic variables included self-reported sex (male or female) and age calculated using their date of birth and the date of survey completion.

Flushing status. Participants answered two questions to indicate whether they: (1) currently experienced facial flushing while drinking alcohol (i.e., “Does your face turn red and feel hot immediately after having a drink?”), or (2) had a tendency to flush in the face during early drinking years (i.e., “Did you have a tendency to flush in the face during the first or second years after you started drinking?”). Those responding ‘yes’ to both questions were categorized as flushers (coded 1) while all others were coded as non-flushers (coded 0). Based on earlier work by Yokoyama et al. [49], these questions show adequate specificity (79%) and high sensitivity (96%) for identifying individuals who carry the inactive ALDH2*2 allele. This indicates that they’re well-suited for screening, especially when the priority is to catch as many true cases as possible, as was prioritized for this study.

Alcohol consumption. We calculated a monthly alcohol consumption variable (grams of ethanol) by multiplying responses to two items: (1) “How often do you have a drink containing alcohol per month?” and (2) “How many drinks containing alcohol do you have on a typical day when you are drinking?” This product was then multiplied by 14, representing the average grams of ethanol per standard drink. Additional details on this measure are provided in [50]. Higher scores indicated greater alcohol consumption.

Drinking motives. Social, conformity, enhancement, and coping motives were measured using the Drinking Motives Questionnaire-Revised [33]. Participants were asked, “How important would you say the following is to you as a reason to drink?” Each motive was measured with a representative item: coping (“because it helps when you feel depressed or nervous”), enhancement (“because it gives me a pleasant feeling”), conformity (“to get in with a group I like”), and social (“because it makes social gatherings more fun”). Responses were recorded on a 4-point Likert scale (1 = strongly disagree to 4 = strongly agree), with higher scores indicating greater endorsement of the respective motive.

Peer drinking. Peer drinking was assessed with a single item, asking participants how many of their college friends got drunk. Response options included none, a few, some, most, or all, coded on a 0–4 scale. Higher scores reflected greater perceived peer drinking.

Current smoking. Current smoking status was assessed with a single item, indicating whether participants smoked cigarettes in the last 30 days. Responses were coded ‘yes’ (coded 1) and ‘no’ (coded 0).

Mental distress. Mental distress was assessed using items from the anxiety and depression subscales of the SCL-90 [51]. Each subscale included four items measuring symptoms experienced in the past week, rated on a 5-point Likert scale (0 = not at all to 4 = extremely). Sample anxiety items included “nervousness or shakiness inside” and “spells of terror or panic,” while sample depression items included “feeling blue” and “feeling no interest in things.” Responses were summed across the eight items for an overall mental distress score (range: 0–32), with higher scores indicating greater distress.

### 2.3. Data Analysis

Statistical analyses were conducted using SPSS (Version 28). We first examined sample characteristics and simple relationships between flushing status and each of the other study variables, using chi-square tests, independent-samples *t* tests, or Mann–Whitney U tests, contingent on the variable’s level of measurement. To assess multivariable relationships, we tested variable main effects in association with alcohol consumption and then whether flushing status moderated associations between each drinking motive and alcohol consumption. Negative binomial regression models were conducted to account for overdispersion exhibited by the outcome variable—alcohol consumption. Analyses included participants with complete data for all variables. Interaction terms were individually added to the model to evaluate their statistical significance. Models stratified by flushing status were conducted to describe the nature of any statistically significant interaction effects.

## 3. Results

### 3.1. Sample Characteristics and Simple Comparisons by Flushing Status

Asian college students who reported alcohol-induced facial flushing were similar to non-flushing students in age, their endorsement of drinking motives, levels of peer drinking and mental distress, current smoking status, and overall alcohol consumption (see Table 1). Conversely, females were overrepresented in the flushing group and underrepresented in the non-flushing group.

On average, flushers consumed fewer standard drinks per month than non-flushers (*M* = 8.8 vs. *M* = 12.9); however, this difference was not statistically significant. Across the total sample, participants were on average 19 years old. They generally reported slight agreement with enhancement and social drinking motives and slight disagreement with coping and conformity motives. On average, they indicated that some of their friends got drunk (response range: none to all), and their mental distress levels were moderately low (scale range: 0–32). Almost 10% of participants were current smokers.

### 3.2. Multivariable Regression Models

Negative binomial models estimated the relationships between alcohol consumption and alcohol-induced flushing, drinking motives, and other related variables. Results are presented in Table 2 in two models. Model 1 included flushing status, drinking motives, and demographic variables. In this model, experiencing facial flushing and greater endorsement of coping motives were associated with lower alcohol consumption, whereas being male and endorsing enhancement and social motives were associated with higher alcohol consumption. Model 2 added peer drinking, mental distress, and current smoking. In this adjusted model, flushing status and coping motives were no longer statistically significant. Higher mental distress was associated with lower alcohol consumption, while enhancement and social motives, male sex, having more peers who got drunk, and being a current smoker were associated with greater alcohol consumption.

### 3.3. Flushing as a Moderator

To test the moderating effect of flushing, interaction terms between flushing status and each drinking motive were added to Model 2, individually. The interaction between flushing and coping was not statistically significant (*B* = 0.174, 95% CI = −0.135, 0.484, *p* = 0.270). However, statistically significant interactions emerged for flushing with enhancement (*B* = 0.849, 95% CI = 0.538, 1.160, *p* < 0.001), conformity (*B* = −0.436, 95% CI = −0.757, −0.114, *p* = 0.008), and social motives (*B* = 0.781, 95% CI = 0.384, 1.178, *p* < 0.001) in their association with alcohol consumption.

Table 3 presents the stratified models for those who did and did not report alcohol-related flushing (flushers and non-flushers), clarifying these interaction effects. Among flushers, greater endorsements of enhancement and social motives were associated with consuming more alcohol, whereas these motives were unrelated to drinking for non-flushers. In fact, none of the drinking motives were associated with alcohol consumption for non-flushers. There was a trend-level effect (*p* < 0.10) between the conformity motive and drinking in both flushers and non-flushers. More specifically, this association between conformity and alcohol consumption was negative for those who flush and positive for those who do not flush. Among the other variables in the model, only perceived peer drinking was associated with alcohol consumption for both flushers and non-flushers, with higher levels of peer drinking linked with increased alcohol intake.

## 4. Discussion

In this sample of Asian young adults in the U.S., we aimed to understand how drinking motives and facial flushing status interrelate in their association with alcohol consumption. Our major finding was that flushing status interacted with three drinking motives, i.e., enhancement, conformity, and social drinking motives, but not with coping-related drinking. More specifically, enhancement and social motives were stronger motives for those who flushed and associated with drinking more alcohol in this group, while conformity motives showed trend-level associations in both groups, suggesting a possible divergence: less drinking among flushers and more among non-flushers. Overall, those who reported flushing or showed higher endorsement of coping motives drank less alcohol, with these relationships attenuated and no longer statistically significant when individuals’ levels of mental distress, perceived peer drinking, and current smoking status were considered. Among these covariates, only peer drinking was associated with more drinking in both flushers and non-flushers. Future research could explore these flushing-motive interactions using daily diary designs (e.g., see [52]) to assess how fluctuations in mental health and social interactions influence drinking motives and substance use behaviors, potentially clarifying causal mechanisms as they unfold in naturalistic settings.

Alcohol-induced facial flushing has long been recognized as a protective factor against alcohol consumption, although not for all college drinkers [15,28,29,53]. Additionally, two converging trends, the higher rates of unhealthy drinking among Asian American youth [3,4,5,6] and the increase in alcohol-intolerant individuals being diagnosed with AUD in international Asian populations [7], underscore the need to examine drinking motives among Asian drinkers who experience facial flushing and to understand how these motives may differ from those who do not flush. To date, studies of facial flushing, drinking motives, and alcohol consumption have been conducted primarily in contexts outside the U.S. The current study is the first one to our knowledge to examine these relationships in an American college sample, contributing to the development of new knowledge in this area for a population recognized as a health disparity group by the NIMHD [2], and offering the potential to inform the development of effective public health promotion efforts.

Although no prior studies have examined interactions between self-reported flushing status and drinking motives, some research has explored interactions between *ALDH2* rs671 genotypes and drinking motives or related constructs. Hendershot et al. [32] found that coping motives were more strongly associated with higher alcohol consumption among individuals without the ALDH2*2 allele, a variant associated with facial flushing and other physiological responses characteristic of alcohol intolerance. Conversely, their study did not find a significant interaction between *ALDH2* genotypes and enhancement motives in relation to alcohol consumption [32]. Notably, Hendershot et al. assessed genotypes rather than self-reported flushing and employed a computer-based task to measure coping and enhancement motives, methodological factors that may account for differences between their findings and those of the current study.

In addition, the sex composition of the Hendershot et al. [32] sample differed from that of the current study; their sample was 55% female compared to 71% in ours. This overrepresentation of females in our study may have influenced the results, particularly given Oh et al.’s [26] finding that female flushers drank more for pleasure and male flushers due to stress or depression. Although epidemiological studies in Asian countries suggest that flushing is more common among males [24], gender may influence self-perception of flushing and tolerance to its effects. Females tend to be more aware of their facial flushing response and report greater embarrassment [54]. Similarly, Newman et al. [55] found notable differences in flushing perception between male and female Chinese students, highlighting the role of gender in how flushing is experienced and interpreted. Importantly, female flushers in that study were more likely to declare the need to stop drinking, and also to intervene in others drinking if they were flushing. Males were less embarrassed by their flushing and less likely to decrease alcohol ingestion and intervene in a peer’s drinking if they were flushing [54,56]. As such, future research should examine how gender identity, socialization, and stigma intersect with physiological alcohol responses to influence drinking motives as well as intervention receptivity.

O’Shea et al. [28] also examined interaction effects using *ALDH2* genotypes and found that peer drinking had a stronger association with alcohol consumption among non-ALDH2*2 carriers than ALDH2*2 carriers. This result is similar to our study’s finding, suggesting that conformity motives were associated with higher alcohol consumption among non-flushers only. Both the O’Shea et al. study and the current analysis used data from the same parent study, albeit different analytic samples, which may partly explain the similarity in results. Yet, Kim et al. [45] indicated that flushers, rather than non-flushers, were more likely to overestimate peer influences and social pressures for drinking greater amounts of alcohol. Because the study by Kim et al. [45], as well as Oh et al. [26], were based on samples outside the U.S., it’s possible that observed differences may reflect cultural variation. This highlights the importance of future research investigating how norms and values shape relationships between drinking motives and alcohol use behaviors among flushers and non-flushers across cultural contexts.

Applying other alternative study designs could further clarify the relationships between drinking motives, flushing, and alcohol consumption. For example, examining patterns or classes of drinking behaviors, as in Iwamoto et al. [44], could improve our understanding of how drinking motives are associated with unhealthy drinking levels among people who flush, given their elevated risks for alcohol-related health harms [18,27]. Notably, the current study found that coping motives were associated with lower alcohol consumption, contrasting with Iwamoto et al.’s [44] finding that coping motives predicted membership in binge drinking classes. Longitudinal study designs, like that used by Greene and Maggs [43], may offer additional insights on how drinking motives change over time for people who flush and could be important for understanding long-term risks for alcohol-related health harms beyond college. The current study’s findings showed that social and enhancement motives were stronger drivers of alcohol use for flushers than non-flushers, aligning with Greene and Maggs [43], who observed increasing effects of social and enhancement motives across college years, with these positive experiences linked to higher drinking frequency and quantity.

The motivational factors behind drinking offer key leverage points for public health efforts to reduce alcohol-related risk. Our findings suggest that prevention strategies could address the unique interplay between physiological sensitivity (e.g., facial flushing) and social or enhancement motives, which were linked to higher alcohol consumption among flushers in this study. Prior results from genetic feedback interventions on alcohol metabolism have shown promising reductions in alcohol consumption among Asian college students [57,58], and could be adapted to address these motives that diminish the protective effect of alcohol-induced facial flushing. Additionally, in the current study, both flushers and non-flushers showed alcohol use patterns influenced by perceived peer drinking, underscoring the value of interventions that address descriptive group norms and impede the modeling of drinking behaviors by peers. Peer-led workshops, student organization collaborations, and culturally informed social marketing campaigns that challenge misperceptions of heavy drinking and drinking motives may therefore have broad impact across Asian student populations [27]. Information on flushing can still serve as a valuable tool for public health guidance, raising awareness of the heightened health risks associated with drinking while flushing, and encouraging safer drinking behaviors aligned with current recommendations for this high-risk group [27]. By combining peer-focused approaches with education on physiological sensitivity, campus prevention programs can better align with the pragmatic needs of public health practice while remaining responsive to Asian students’ diverse experiences. Finally, campus health services might consider voluntary screening for alcohol-induced flushing and drinking motives during wellness visits or alcohol education programs, as assessing both physiological responses and motivational drivers could help identify students at risk who might not otherwise meet criteria for problematic use based solely on drinking frequency or quantity.

It is important to interpret these findings in light of both study strengths and limitations. First, the study’s novelty is a key strength, as it is one of the few investigations to examine reasons for drinking among Asian college students in the U.S. with flushing reactions [12]. Most prior research has focused on international samples (e.g., [26,45,55]), highlighting the value of replication in U.S. contexts. One limitation is that our sample included Asian students from diverse backgrounds rather than being limited to East Asian ancestry, which is more strongly associated with genetic variants linked to alcohol intolerance and flushing [e.g., ALDH2*2 (rs671) and ADH1B*2 (rs1229984)]. However, this diversity is also a strength, as it allows for broader generalizability to the heterogeneous Asian student population on U.S. campuses. Another limitation is the sample was majority female, which may reduce the applicability of findings to males, especially given reports of gender-specific responses to flushing [26,56]. However, this higher female representation allowed us to capture gender patterns relevant to Asian college women, an underexamined group in alcohol research.

Additionally, facial flushing was assessed via self-report, without distinguishing between “fast flushers” and “slow flushers” [59]. Nonetheless, self-reported flushing has been validated as a reliable indicator of alcohol intolerance [49], supporting the credibility of this approach. Another limitation is that drinking motives were assessed using a single item for each motive rather than full subscales, which may limit measurement precision. However, prior research demonstrates that single-item measures can reliably capture core dimensions of drinking motives, particularly in large-scale or time-sensitive studies [60]. Finally, acculturation and U.S. birthplace were not measured across all cohorts, despite prior research showing these variables influence unhealthy drinking among Asians in the U.S. [43,44]. However, by focusing on students enrolled at a U.S. university, the study captured individuals embedded in an American college drinking culture, where acculturation-related influences are highly relevant.

## 5. Conclusions

This study provides an understanding of how drinking motives interact with facial flushing status to influence alcohol consumption among Asian young adults at a U.S. college. Our findings reveal that while conformity motives indicated greater alcohol use among non-flushers, enhancement and social motives were more strongly associated with drinking among individuals who flush. Notably, flushing status was less protective after accounting for mental distress, peer drinking, and current smoking, underscoring the relevance of psychosocial context. These results highlight the interrelationships involved in alcohol-related decision-making among those with physiological sensitivity and call for the use of motive- and peer-based prevention strategies. By tailoring genetic feedback interventions to account for motivational factors, universities may better support the health and well-being of their Asian students who flush. Future research should continue to explore these intersections through longitudinal or context-sensitive designs and focus more on unhealthy drinking behaviors (e.g., binge drinking) to inform effective public health alcohol harm-reduction efforts for people who drink despite facial flushing.

## Figures and Tables

**Table 1:** Characteristics of *N* = 244 Asian college students by flushing status.

	Total Sample	Alcohol-Related Flushing		
	% or *M* (*SD*)	Yes	No	*p*-Value
Flushing (yes)	34.0%	--	--	--
Female	70.5%	80.7%	65.6%	**0.014**
Age	19.4 (0.5)	19.5 (0.5)	19.4 (0.5)	0.183
Coping motive *	2 (2)	2 (2)	2 (2)	0.125
Enhancement motive *	3 (1)	3 (1)	3 (1)	0.931
Conformity motive *	2 (2)	1 (2)	2 (2)	0.554
Social motive *	3 (1)	3 (0)	3 (1)	0.289
Peer drinking *	2 (2)	2 (2)	2 (2)	0.983
Mental distress	8.7 (7.1)	9.7 (8.0)	8.1 (6.5)	0.319
Current smoking	9.9%	10.8%	9.4%	0.716
Alcohol consumption	162.8 (345.2)	123.4 (291.0)	180.9 (369.5)	0.400

Note: * Levels of endorsement for drinking motives and peer drinking are ordinal—the median (Mdn) and interquartile range (IQR) are presented [Mdn (IQR)]; statistically significant differences (*p* < 0.05) between students reporting flushing (yes vs. no) are bold.

**Table 2:** Main effects model * of alcohol consumption: Examining flushing status, drinking motives, and covariates.

	Model 1		Model 2	
	*B* (95% CI)	*p*-Value	*B* (95% CI)	*p*-Value
Flushing (ref. no)	**−0.43 (−0.72, −0.14)**	**0.003**	−0.13 (−0.44, 0.19)	0.425
Male (ref. female)	**0.76 (0.46, 1.05)**	**<0.001**	**0.66 (0.31, 1.00)**	**<0.001**
Age	−0.001 (−0.27, 0.27)	0.995	−0.03 (−0.29, 0.24)	0.856
Coping	**−0.16 (−0.30, −0.01)**	**0.032**	−0.07 (−0.24, 0.09)	0.373
Enhancement	**0.22 (0.05, 0.38)**	**0.010**	**0.38 (0.20, 0.56)**	**<0.001**
Conformity	0.05 (−0.11, 0.22)	0.51	−0.01 (−0.17, 0.15)	0.891
Social	**0.49 (0.28, 0.70)**	**<0.001**	**0.38 (0.17, 0.59)**	**<0.001**
Peer drinking	--	--	**0.41 (0.30, 0.52)**	**<0.001**
Mental distress	--	--	**−0.03 (−0.05, −0.01)**	**0.009**
Current smoking	--	--	**1.28 (0.74, 1.82)**	**<0.001**

Note: * Negative binomial models were estimated to account for overdispersion exhibited by the outcome variable; statistically significant effects (*p* < 0.05) are bold.

**Table 3:** Stratified main effects model * of alcohol consumption: Asian students who flush (*n* = 83) and non-flushers (*n* = 161).

	Flushers		Non-Flushers	
	*B* (95% CI)	*p*-Value	*B* (95% CI)	*p*-Value
Male (ref. female)	0.49 (−0.11, 1.08)	0.108	**0.44 (0.04, 0.83)**	**0.029**
Age	**−0.70 (−1.17, −0.22)**	**0.004**	0.15 (−0.20, 0.49)	0.399
Coping	−0.18 (−0.45, 0.10)	0.218	0.01 (−0.20, 0.22)	0.909
Enhancement	**0.77 (0.48, 1.07)**	**<** **0.001**	0.10 (−0.14, 0.34)	0.433
Conformity	−0.24 (−0.51, 0.04)	0.090	0.20 (−0.003, 0.40)	0.054
Social	**0.52 (0.15, 0.89)**	**0.006**	0.16 (−0.11, 0.42)	0.248
Peer drinking	**0.22 (0.09, 0.40)**	**0.016**	**0.72 (0.57, 0.87)**	**<** **0.001**
Mental distress	0.02 (−0.02, 0.05)	0.358	**−0.05 (−0.07, −0.02)**	**0.001**
Current smoking	0.11 (−0.66, 0.88)	0.780	**1.50 (0.80, 2.21)**	**<0.001**

Note: * Negative binomial models were estimated to account for overdispersion exhibited by the outcome variable; statistically significant effects (*p* < 0.05) are bolded.

## Data Availability

Data from this study are available via spit4science@vcu.edu to qualified researchers who provide the appropriate signed data use agreement.

## Abbreviations

National Institute on Minority Health and Health Disparities: NIMHD; Alcohol Use Disorder: AUD; Aldehyde Dehydrogenase 2*2 variant: *ALDH2**2; Alcohol Dehydrogenase 1B2 variant: *ADH1B**2; Fear of Missing Out: FOMO; United States: U.S.; Research Electronic Data Capture: REDCap; Symptom Checklist-90: SCL-90; Statistical Package for the Social Sciences: SPSS; Median: Mdn; Interquartile Range: IQR; Confidence Interval: CI.
